# Systematic screening for advanced liver fibrosis in patients with coronary artery disease: The CORONASH study

**DOI:** 10.1371/journal.pone.0266965

**Published:** 2022-05-26

**Authors:** Thierry Thévenot, Sophie Vendeville, Delphine Weil, Linda Akkouche, Paul Calame, Clémence M. Canivet, Claire Vanlemmens, Carine Richou, Jean-Paul Cervoni, Marie-France Seronde, Vincent Di Martino, Jérôme Boursier

**Affiliations:** 1 Department of Hepatology, University Hospital Jean Minjoz, Besançon, France; 2 Department of Cardiology, University Hospital Jean Minjoz, Besançon, France; 3 Department of Radiology, University Hospital Jean Minjoz, Besançon, France; 4 Hepato-Gastroenterology Department, Angers University Hospital, Angers, France; Osaka City University Graduate School of Medicine, JAPAN

## Abstract

Although coronary artery disease (CAD) and advanced liver fibrosis (AdLF) are commonly associated in patients with non-alcoholic fatty liver disease (NAFLD), the prevalence of AdLF and the diagnostic performance of non-invasive fibrosis tests (NITs) in CAD patients remains unknown. We aimed to prospectively screen for AdLF in patients with documented CAD using NITs and Fibroscan. High and intermediate zones of NITs were combined to define AdLF. AdLF was suspected whenever APRI ≥ 0.5, Forns index ≥ 4.2, NAFLD fibrosis score (NFS) ≥ -1.455/0.12 for age </≥ 65 yrs), Fib4 (≥ 1.30/2.0 for age </≥ 65 yrs) and eLIFT≥ 8. A presumed AdLF assessed by Fibroscan ≥ 8 kPa was the primary outcome measure. Results were given on the basis of intent-to-diagnose liver stiffness ≥ 8 kPa. Among 189 patients (age 60±7years), 10 (5.3%) had a Fibroscan ≥ 8 kPa, of whom 5 underwent liver biopsy (F3/F4: n = 3; no fibrosis: n = 2). AdLF was suspected in 31% of cases using eLIFT (specificity, Sp 70%), 85% with Forns (Sp 16%), 38% with NFS (Sp 63%), 25% with Fib4 (Sp 74%), and 10% with APRI (Sp 91%). In 149 patients “at-risk” of NAFLD (i.e., elevated ALT or diabetes or hypertriglyceridemia or BMI ≥25 kg/m^2^), AdLF ranged between 10% (APRI) to 84% (Forns). In this subgroup, the most efficient NITs to predict Fibroscan ≥ 8 kPa were eLIFT (Se 60%, Sp 70%) and NFS (Se 70%, Sp 60%). Finally, in CAD patients with risk factors for NAFLD, NFS or the more user-friendly eLIFT are the most attractive first-line biochemical NITs to discriminate good candidates for Fibroscan.

## Introduction

Nonalcoholic fatty liver disease (NAFLD) is the most prevalent chronic liver disease, affecting 25% of the worldwide population [1]. NAFLD includes a spectrum of diseases ranging from simple fatty liver to nonalcoholic steatohepatitis (NASH), and this latter may progress silently to cirrhosis and hepatocellular carcinoma. NAFLD is associated with cardiovascular disease (CVD) due to shared metabolic risk factors (obesity, dyslipidemia, diabetes and arterial hypertension), but also independently of these risk factors [2]. Liver fibrosis is the most important prognostic factor in patients with NAFLD [3, 4] and liver biopsy is considered as the gold standard for evaluating the extent of fibrosis. However, liver biopsy presents unavoidable limitations, including its invasiveness associated with potentially severe complications, poor acceptability, inter/intra-observer variability and cost. Because of these limitations, non-invasive fibrosis tests (NITs) have been developed to identify patients with advanced liver fibrosis (AdLF) and monitor progression of fibrosis over time [5, 6]. Many studies have shown that NITs are accurate tools for the case-finding of patients with liver fibrosis in the general population or in populations with liver risk factors [7]. However, although patients with CAD have increased metabolic and thus, liver-risk factors, no work has evaluated NITs in this population. CVD remains the leading cause of morbidity and mortality in patients with NAFLD and NASH [8] and several recent studies have confirmed a significant association between AdLF as assessed by NITs or liver biopsy, and cardiovascular outcomes [8–11]. The 2016 European clinical practice guidelines for the management of NAFLD strongly recommend CVD risk assessment in all patients with NAFLD, regardless of the presence of traditional risk factors [5]. Conversely, NAFLD screening in patients with coronary artery disease (CAD) has not been clearly indicated, although atherosclerosis is a feature shared by both conditions, and the progression of hepatic fibrosis represents a major risk factor for hepatocellular carcinoma and liver disease-related death. The “real-life” prevalence of AdLF and the results of NITs in patients with CAD have never been evaluated to date. Therefore, the present CoroNASH study aimed to report the first experience of systematic screening for AdLF in patients with proven CAD, with the following specific objectives: 1) to determine the prevalence of AdLF, as measured by Fibroscan, and 2) to evaluate the diagnostic performance of first-line biochemical NITs for detecting AdLF in these patients.

## Materials and methods

### Patients

All consecutive patients aged 18 to 70 years, admitted to the Cardiology Department of the University Hospital of Besançon from May 2019 to March 2020 for scheduled coronary angiography were screened for eligibility, and were confirmed eligible for participation if coronary stenosis was found on the coronary angiography. The upper age threshold of 70 years was arbitrarily chosen for pragmatic clinical purposes, considering that liver transplantation is often denied to these patients. We excluded patients who met any one or more of the following criteria: presence of implantable medical device (defibrillator or pacemaker), right ventricular heart failure or a left ventricular ejection fraction < 40%, prothrombin time (or factor V for patients receiving anticoagulants) < 70% or serum bilirubin ≥ 30 μmol/L (criteria suggesting advanced liver fibrosis), known liver disease or ascites, extra-hepatic cholestasis, pregnant women and thrombocytopenia associated with hematologic disorder.

This study was conducted according to the principles of the Declaration of Helsinki and written informed consent was obtained from all participants. The current research study was considered as a routine clinical care since Fibroscan is usually performed in CAD patients to assess liver fibrosis in our university hospital and NITs were calculated on biological variables carried out routinely by cardiologists. According to the French Regulatory Authority for clinical studies, the CORONASH study received no opposition from the local ethics committee East Area II.

### Data collection and definitions

Patients who met the inclusion criteria had a short study visit to collect demographic data and undergo physical examination. The following characteristics were recorded: age, gender, body mass index (BMI), alcohol and tobacco consumption within the previous 12 months, use of antidiabetic, antihypertensive, and lipid-lowering medications. A 12-hour fasting blood sample was drawn for all patients on the day of coronary angiography, and liver function tests, glucose, lipids, and hepatitis B and C markers were analysed. Dyslipidemia was defined by hypertriglyceridemia > 1.7 mmol/L or serum HDL cholesterol < 1.0/1.3 mmol/L for men/women respectively, or use of specific treatment for lipid abnormalities. Metabolic syndrome was defined according to the International Diabetes Federation group [12]; briefly, a waist circumference ≥94/≥80 cm for men/women with any two of the following factors: elevated blood pressure, elevated glycaemia, elevated triglycerides or reduced HDL cholesterol. Average daily alcohol consumption was estimated over the past 12 months and a standard drink was defined as 10 g of ethanol. Excessive drinking was considered when alcohol use exceeded 20 g/day for women and 30 g/day for men.

### First-line biochemical non-invasive fibrosis tests

Five non-patented NITs for liver fibrosis, which have been applied in patients with chronic liver disease of different aetiologies [13, 14], were calculated: AST-to platelet ratio index (APRI) [15], Fibrosis-4 (FIB-4) [16], Forns index [17], NAFLD fibrosis score (NFS) [18] and eLIFT [19] (S1 File). We categorized patients into low-, intermediate- and high-risk groups for AdLF based on the following suggested (lower and upper) cutoffs: APRI (0.5 and 1.5), FIB-4 (lower cutoff 1.30 if age <65, and 2.0 if age ≥65 years; upper cutoff 2.67) [20], Forns (4.2 and 6.9), NFS (lower cutoff -1.455 if age <65, and 0.12 if age ≥65 years; upper cutoff 0.676) [20]. The eLIFT was considered negative when the score was < 8 and positive when ≥ 8.

### Coronary angiography

Patients underwent invasive coronary angiography on the morning of admission and coronary fluoroscopic images were interpreted by MFS who has more than 20 years of reading experience. CAD was defined as any degree of stenosis on any of the coronary arteries by coronary angiography [21]. Indications for coronary angiography were silent ischemia, stable angina, heart failure, for initial evaluation of ischemic heart disease, or prior to cardiac surgery.

### Fibroscan

All patients had a Fibroscan^®^ (Echosens, Paris, France) on the same day as coronary angiography, in a fasting condition (at least two hours) for liver stiffness measurement (LSM), expressed in kilopascals (Kpa) and to diagnose steatosis using the controlled attenuation parameter (CAP in dB/m). Fibroscan was performed by two experienced operators (SV and a dedicated nurse) by using an M probe (or XL probe when BMI >30 kg/m^2^ or LSM failure with M probe) as per the EASL-ALEH clinical practice guidelines [14]. LSM was considered reliable if the operator obtained a minimum of 10 valid measurements together with an interquartile range ≤30% of the median value of LSM (IQR/median of LSM ≤0.3). The cut-off point of CAP to define the presence of hepatic steatosis was set at 275 dB/m, as recently suggested [22]. We used the same LSM cut-off (< 8 kPa) when M or XL were used, according to the appropriate BMI to confidently exclude AdLF [23–25].

### Liver biopsy

A transjugular liver biopsy (TLB) was proposed for patients with LSM ≥ 8 kPa, as previously suggested [13]. TLB was planned six months later, when the dual antiplatelet therapy was scaled back to single antiplatelet therapy. Liver biopsy specimens were fixed in formalin, embedded in paraffin and stained with picrosirius red to highlight collagen networks. Slides were analyzed independently by one experienced pathologist who was blinded to the results of Fibroscan and NITs. Liver fibrosis was evaluated according to NASH CRN staging in patients with NAFLD, and METAVIR staging in other patients.

### Statistical analysis

Quantitative variables are presented as mean ± standard deviation (SD) or as median with interquartile range (IQR), and categorical variables as number (percentage). Continuous variables were compared using the Student *t*-test or the Mann-Whitney test, and categorical variables using the Chi-squared test or Fisher’s exact test, as appropriate. The performance of NITs for the diagnosis of LSM ≥ 8 kPa was assessed by receiver operating characteristic (ROC) curve analysis. The area under the ROC (AUROC) curve was used to compare accuracy of NITs. Sensitivity, specificity, positive predictive value (PPV), negative predictive value (NPV), and accuracy of NITs were calculated. We have no missing data for the LSM and the rare missing data for NITs have been ignored. Continuous variables influencing LSM in univariate analysis were dichotomized according to their optimal threshold value (using the Youden’s index) and were analyzed by multivariate logistic regression with backward selection. Analyses were performed on the whole population and in subgroups of patients with a higher risk of NAFLD, as recently done by others [26]. To this end, patients were stratified into groups “at risk” of NAFLD. (i.e., patients with any of the following risk factors: elevated ALT [defined by our local upper limit of normal, 55 IU/L], diabetes, dyslipidemia or BMI ≥25 kg/m^2^) and at “high-risk” of NAFLD (this group differed only by a BMI ≥30 kg/m^2^). According to the cut-offs of FIB-4, APRI, Forns, and NFS, patients were categorized into low-, intermediate- and high-risk categories for AdLF. For all analyses, we combined the intermediate- and high-risk groups to avoid missing patients with suspected AdLF. A 95% confidence interval (CI) for each statistic was calculated from a binomial distribution. A p value <0.05 was considered statistically significant. All statistical analyses were performed using NCSS for Windows (2010).

## Results

### Study population

**Fig 1:**
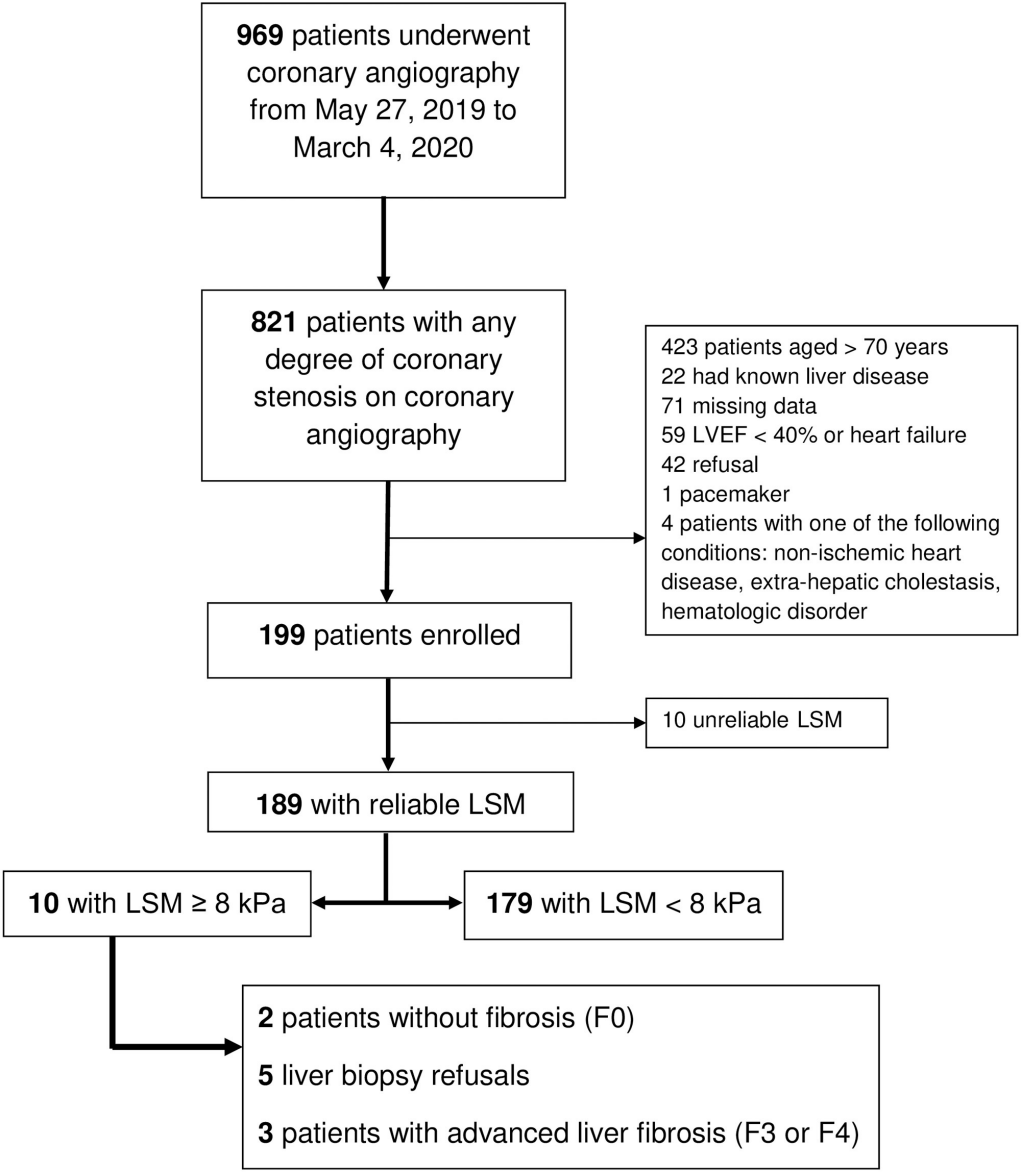
Flowchart of the CoroNASH study. LSM, liver stiffness measurements; LVEF, left ventricular ejection fraction.

From May 2019 through March 2020, 969 patients underwent coronary angiography for the following indications: silent ischemia (n = 247), stable angina (n = 241), ischemic heart disease (n = 207), heart failure (n = 96) and before corrective cardiac surgery (n = 174). A flow-chart of the study population is shown in Fig 1. The main reason for exclusion was age more than 70 years. A final total of 199 patients were included in the present study. The baseline clinical and biological characteristics of the patients are detailed in Table 1. The mean age of the study population was 60.5±7.5 years, 80.9% were male, 57.4% had metabolic syndrome and, 71.9% were overweight or obese. NAFLD, suspected in the presence of a CAP ≥275 dB/m, was present in 46.2% of our cohort. Only 12 patients drank excessively and only one of them had LSM ≥ 8 kPa (alcohol consumption 60 g/day) together with metabolic syndrome. All patients were seronegative for hepatitis B surface antigen and antibodies to hepatitis C, 6 patients had both anti-HBc and anti-HBs antibodies and only one carried isolated anti-HBc antibodies. No patients carrying anti-HBc antibodies had LSM ≥ 8 kPa. All patients with suspected AdLF had a Hepatology visit that ruled out some specific chronic liver diseases (S2 File).

**Table 1:** Biological and demographic characteristics of patients.

Variables	Results
**Age, years**	60.5 ± 7.3
**Men, n (%)**	153 (80.9)
**BMI, kg/m** ^**2**^	27.6 ± 4.3
**BMI ≥ 30 kg/m**^**2**^, **n (%)**	47 (24.9)
**BMI ≥ 25 kg/m**^**2**^, **n (%)**	135 (71.4)
**Smokers, n (%)**	126 (66.7)
**Diabetes, n (%)**	54 (29.6)
**Arterial hypertension, n (%)**	139 (73.5)
**Dyslipidemia, n (%)**	170 (90.0)
**Metabolic syndrome, n (%)**	101 (56.1)
**Patients at “high-risk” of NAFLD**^†^, **n (%)**	104 (55,3)
**Patients “at risk” of NAFLD**^‡^, **n (%)**	149 (79.6)
**Alcohol consumption, g/d**	0 [0–15]
**Excessive alcohol consumption, n (%)**	12 (6.5)
**Glycosylated hemoglobin, %**	6.30 ± 0.97
**HDL cholesterol, mmol/L**	1.04 ± 0.26]
**LDL cholesterol, mmol/L**	2.23 ± 0.91
**Total cholesterol, mmol/L**	3.72 ± 1.17
**Triglycerides, mmol/L**	1.49 ± 0.82
**Aspartate aminotransferase, IU/L**	28.3 ± 12.4
**Alanine aminotransferase, IU/L**	32.7 ± 26.2
**Gamma-glutamyl transferase, IU/L**	30 [22–50]
**Alkaline phosphatase, IU/L**	71 [57–88]
**Total bilirubin, μmol/L**	13.0 ± 6.8
**Albumin, g/L**	36.0 ± 2.8
**Platelets, G/L**	256 ± 72
**Creatinine, μmol/L**	83.9 ± 23.4
**C-reactive protein, mg/L**	1.3 [0.7–2.9]
**Prothrombin index or Factor V, %**	95.8 ± 11.7
**CAP ≥ 275 dB/m, n (%)**	88 (47.0)

Quantitative data are expressed as mean ± standard deviation (SD) or median [interquartiles; IQR].

^†^: Patients with at least one of the following conditions: elevated ALT, diabetes, triglycerides ≥ 1.7 mmol/L, BMI ≥ 30 kg/m^2^.

^‡^: Patients with at least one of the following conditions: elevated ALT, diabetes, triglycerides ≥ 1.7 mmol/L, BMI ≥ 25 kg/m^2^.

BMI, body mass index; CAP, controlled attenuation parameter; HDL, high-density lipoprotein; LDL, low-density lipoprotein.

Among the 10 patients with LSM ≥ 8 kPa, 8 had metabolic syndrome and 4 had NAFLD, as suggested by CAP ≥275 dB/m. The group “at risk” of NALFD included 158 patients (overweight 90.5%, hypertriglyceridemia 36.1%, elevated ALT 10.1%, and diabetes 37.5%) and the group “at high-risk” of NAFLD included 111 patients (obese 47.7%, hypertriglyceridemia 51.3%, elevated ALT 14.4%, and diabetes 53.1%).

### Fibroscan

Of 199 patients evaluated using Fibroscan, 10 (5%) had unreliable results (IQR/median of LSM >0.3) of whom six with an XL probe and four with an M probe. Therefore, the final study population for the analysis using Fibroscan consisted of 189 patients. The XL probe was used in 62 patients (32.8%, 47 obese and 15 M probe failures), and the M probe in 127 patients (67.2%). The median age (62 *vs* 60 years; p = 0.23), proportion of men (81.1 *vs* 80.6%, p = 0.94) and LSM (4.9 *vs* 5.3 kPa; p = 0.60) did not differ significantly between patients evaluated using M and XL probes, respectively. Among the 189 patients with a valid LSM, AdLF (LSM ≥8 kPa) was suspected in 10 patients (five using the M probe and five the XL probe). The estimated prevalence of AdLF was thus 5.3% (95%CI: 2.1%-8.5%) in our population. Steatosis (CAP ≥ 275 dB/m) was detected in 87 patients (46.5%), and among these, LSM ≥ 8 kPa was observed in 4 (4.6%; 95%IC: 0.2%-9.0%).

LSM was higher in patients with metabolic syndrome (5.47±2.01 *vs* 4.92±1.34 kPa in patients without metabolic syndrome; p = 0.062), in those with elevated ALT (6.54±2.80 *vs* 5.15±1.58 kPa; p = 0.032) and in those with GGT ≥ 30 IU/L (median value in patients with a valid Fibroscan; 5.69 ±1.77 *vs* 4.82±1.63 kPa; p = 0.0001). LSM did not differ significantly according to obesity (p = 0.14), diabetes (p = 0.42), hypertriglyceridemia (p = 0.37), or NAFLD defined as CAP ≥275 dB/m (p = 0.15). Compared to patients with LSM < 8 kPa, those with LSM ≥8 kPa had higher ALT (p = 0.05) and GGT (p = 0.074), had lower HDL (p = 0.01) and prothrombin time/factor V (p = 0.09) (Table 2). By multivariate analysis, the factors associated with presence of LSM were HDL cholesterol and GGT (S1 Table).

**Table 2:** Biological and demographic characteristics of patients according to liver stiffness.

Variables	LSM ≥ 8 kPa N = 10	LSM < 8 kPa N = 179	P
**Age, years**	63.7 ± 3.6	60.4 ± 7.4	0.23
**Men, n (%)**	9 (90)	144 (80.4)	0.74
**BMI, kg/m** ^**2**^	29.4 ± 5.8	27.5 ± 4.2	0.15
**BMI ≥ 30 kg/m**^**2**^, **n (%)**	4 (40)	43 (24.0)	0.45
**BMI ≥ 25 kg/m**^**2**^, **n (%)**	9 (90)	126 (70.4)	0.32
**Smokers, n (%)**	5 (50)	121 (67.6)	0.42
**Diabetes, n (%)**	5 (50)	49 (27.4)	0.23
**Arterial hypertension, n (%)**	7 (70)	132 (73.7)	0.73
**Dyslipidemia, n (%)**	9 (90)	161 (89.9)	1.00
**Metabolic syndrome, n (%)**	8 (80)	93 (54.7)	0.19
**Patients at “high-risk” of NAFLD**^†^, **n (%)**	8 (80)	96 (53.9)	0.19
**Patients “at risk” of NAFLD**^‡^, **n (%)**	10 (100)	139 (78.1)	0.21
**Alcohol consumption, g/jr**	0 [0–30]	0 [0–10]	0.61
**Excessive drinker, n (%)**	1 (10)	11 (5.9)	0.50
**Glycosylated hemoglobin, %**	6.6 ± 1.0	6.28 ± 0.97	0.22
**HDL cholesterol, mmol/L**	0.85 ± 0.27	1.05 ± 0.27	0.01
**LDL cholesterol, mmol/L**	2.1 ± 0.9	2.24 ± 0.91	0.61
**Total cholesterol, mmol/L**	3.6 ± 1.2	3.7 ± 1.2	0.55
**Triglycerides, mmol/L**	1.71 ± 0.86	1.48 ± 0.82	0.28
**Aspartate aminotransferase, IU/L**	27.5 [22.2–57.5]	26 [22–30.2]	0.062
**Alanine aminotransferase, IU/L**	48.5 [22.7–97]	27 [19–36]	0.050
**Gamma-glutamyl transferase, IU/L**	53 [26–132]	30 [22–48]	0.074
**Alkaline phosphatase, IU/L**	56 [40–84.7]	72 [58–89]	0.10
**Total bilirubin, μmol/L**	12 ± 3.4	13.1 ± 6.9	0.94
**Albumin, g/L**	36.4 ± 2.8	36.0 ± 2.8	0.56
**Platelets, G/L**	226 ± 43	258 ± 73	0.20
**Prothrombin index or Factor V, %**	89.3 ± 11.7	96.2 ± 11.6	0.09
**Creatinine, μmol/L**	89.3 ± 24.4	83.6 ± 23.4	0.34
**C-reactive protein, mg/L**	2 [0.57–4.57]	1.3 [0.7–2.9]	0.59
**CAP ≥ 275 dB/m, n (%)**	4 (40)	83 (46.9)	0.92
**Fibroscan, kPa**	10.0 ± 1.9	5.0 ± 1.3	<0.0001

Quantitative data are expressed as mean ± standard deviation (SD) or median [interquartiles; IQR].

^†^: Patients with at least one of the following conditions: elevated ALT, diabetes, triglycerides ≥ 1.7 mmol/L, BMI ≥ 30 kg/m^2^.

^‡^: Patients with at least one of the following conditions: elevated ALT, diabetes, triglycerides ≥ 1.7 mmol/L, BMI ≥ 25 kg/m^2^.

BMI, body mass index; CAP, controlled attenuation parameter; HDL, high-density lipoprotein; LDL, low-density lipoprotein; LSM, Liver stiffness measurement.

### Non-invasive fibrosis test results in the whole population

The proportion of patients in the low, intermediate and high-risk zones is shown in S2 Table. A detailed summary of the sensitivity, specificity, PPVs, NPVs and the AUROC for AdLF (LSM ≥8.0 kPa) of the five NITs is shown in Table 3. AUROCs of FIB-4 and APRI were 0.647 and 0.678, respectively. Only 2 and 3 patients with LSM ≥8 kPa were identified using FIB-4 and APRI, respectively. Although the Forns index showed good performance (AUROC 0.728), its 100% sensitivity implies that too many false-positive cases (79.4%) could potentially receive unnecessary additional evaluations. Excluding Forns, NPVs of the other NITs ranged between 94.3% (APRI) and 97.4% (NFS). The proportion of patients referred for LSM evaluation after first-step testing with NITs (excluding Forns) ranged between 10.1% (APRI) and 38.5% (NFS). NFS had good sensitivity (70%) but failed to identify one patient with histological AdLF. The eLIFT score had a high NPV (only 2.1% of false-negatives). Results regarding NITs, LSM and TLB are summarized in S1 Fig and show that eLIFT and NFS are of potential interest in this CAD population.

**Table 3:** Screening for advanced liver fibrosis (LSM ≥8.0 kPa) using non-invasive fibrosis tests in patients with a valid Fibroscan.

Tests	N	Cut-off	Se % (95% CI)	Spe % (95% CI)	PPV % (95% CI)	NPV % (95% CI)	Accuracy %	AUROCs (95% CI)	Patients with suspected AdLF on NITs^&^, n (%)	LSM ≥ 8 kPa among H-I NITs^&^, n (%)
**NFS**	187	≥-1.455/0.12^†^	70.0 (39.7–89.2)	63.3 (55.9–70.0)	9.7 (5.5–13.9)	97.4 (95.1–99.7)	63.6 (60.1–67.2)	0.686 (0.466–0.826)	72 (38.5)	7 (9.7)
**Forns**	189	≥4.2	100 (72.2–100)	16.2 (11.5–22.3)	6.2 (2.7–9.7)	100 (1–1)	20.6 (17.6–23.5)	0.728 (0.536–0.848)	160 (84.6)	10 (6.2)
**APRI**	188	≥0.5	30.0 (10.8–60.3)	91.0 (85.9–94.4)	15.8 (10.6–21.0)	95.8 (92.9–98.6)	87.7 (85.3–90.1)	0.678 (0.386–0.846)	19 (10.1)	3 (15.8)
**FIB-4**	188	≥1.30/2.0^†^	20.0 (5.7–51.0)	74.2 (67.3–80.0)	4.2 (1.3–7.1)	94.3 (91.0–97.6)	71.3 (68.0–74.6)	0.647 (0.473–0.772)	48 (25.5)	2 (4.2)
**eLIFT**	189	≥8	60.0 (31.3–83.2)	70.4 (63.3–76.6)	10.2 (5.9–14.5)	96.9 (94.4–99.4)	69.8 (66.5–73.1)	0.739 (0.543–0.858)	59 (31.2)	6 (10.2)

^&^: High and intermediate zones (H-I zones) of NFS, APRI, Forns and FIB-4 were grouped; patients in the H-I zones and those with eLIFT ≥ 8 had suspected AdLF and were referred for liver stiffness measurement (LSM).

^†^: First cut-off for patients aged < 65 years, second cut-off for patients aged ≥65 years.

NITs, non-invasive fibrosis tests.

### Non-invasive fibrosis tests in subgroups according to the risk of NAFLD

The performances of NITs were evaluated in 149 (78.8%) patients “at risk” of NAFLD with valid LSMs (Table 4). In this subgroup, 10 patients had AdLF as assessed by LSM ≥8. APRI, FIB4 and Forns showed the same limitations as in the overall population: APRI and FIB-4 had low sensitivity (30% and 20%, respectively) and a high number of patients with AdLF were not identified (7/10 with APRI and 8/10 with FIB-4). Conversely, Forns recognized all AdLF thanks to 100% sensitivity, but with a very low specificity (17.3%) that renders Fibroscan mandatory for the majority of patients (84%). NFS and eLIFT had similar NPV and performance (AUROC 0.662 *vs* 0.743 for eLIFT; p = 0.55). The proportion of “at-risk” patients referred for LSM evaluation after first-step testing with eLIFT and NFS was substantially reduced to 31.5% and 42.2%, respectively. Suspected AdLF was not detected in one and two patients with NFS and eLIFT, respectively. Among the six patients with both eLIFT >8 and LSM ≥8 kPa, four consented to undergo liver biopsy, and AdLF was histologically confirmed in three of these four patients. Results regarding NITs, LSM and TLB are summarized in S2 Fig.

**Table 4:** Screening for advanced liver fibrosis (LSM ≥8.0 kPa) using non-invasive fibrosis tests in the “at risk” subgroup of NAFLD with a valid fibroscan.

Tests	N^‡^	Cut-off	Se % (IC 95%)	Spe % (IC 95%)	PPV % (IC 95%)	NPV % (IC 95%)	Accuracy %	AUROC (IC 95%)	Patients with suspected AdLF on NITs^&^, n (%)	LSM ≥ 8 kPa among H-I NITs^&^, n (%)
**NFS**	147	≥-1.455/0.12^†^	70.0 (39.7–89.2)	59.8 (51.5–67.7)	11.3 (6.2–16.4)	96.5 (93.5–99.5)	60.5 (56.5–64.5)	0.662 (0.446–0.805)	62 (42.2)	7 (11.3)
**Forns**	149	≥4.2	100 (72.2–100)	17.3 (11.9–24.4)	8.0 (3.6–12.4)	100 (1–1)	22.8 (19.4–26.2)	0.738 (0.555–0.853)	125 (83.9)	10 (8.0)
**APRI**	148	≥0.5	30.0 (10.8–60.3)	91.3 (85.4–94.9)	20.0 (13.5–26.4)	94.7 (91.1–98.3)	87.2 (84.5–89.9)	0.707 (0.451–0.855)	15 (10.1)	3 (20.0)
**FIB-4**	148	≥1.30/2.0^†^	20.0 (5.7–51.0)	77.5 (69.9–83.7)	6.0 (2.2–9.8)	93.0 (88.9–97.1)	73.6 (70.0–77.2)	0.687 (0.518–0.804)	33 (22.3)	2 (6.1)
**eLIFT**	149	≥8	60.0 (31.2–83.2)	70.5 (62.5–77.4)	12.7 (7.3–18.0)	96.1 (93.0–99.2)	69.8 (66.0–73.6)	0.743 (0.550–0.860)	47 (31.5)	6 (12.8)

^&^: High and intermediate zones (H-I zones) of NFS, APRI, Forns and FIB-4 were grouped; patients in the H-I zones and those with eLIFT ≥ 8 had a suspected advanced liver fibrosis and were referred for liver stiffness measurement (LSM).

^‡^: Patients with at least one of the following conditions: elevated ALAT (n = 16, 10.7%), diabetes (n = 54, 36.2%), triglycerides ≥ 1.7 mmol/L (n = 55, 36.9%), BMI ≥ 25 kg/m^2^ (n = 135, 90.6%).

^†^: First cut-off for patients aged < 65 years, second cut-off for patients aged ≥65 years.

NITs, non-invasive fibrosis tests.

We also evaluated performances of NITs in 104 “high-risk” patients with a valid Fibroscan (S3 Table). In this subgroup, only eight patients had LSM ≥8 kPa, meaning that AdLF was missed in two overweight, but not obese patients. As in the “at-risk” subgroup, we observed high sensitivity (100%) of the Forns index, contrasting with low sensitivity for APRI (25%) and FIB-4 (37.5%). Using NFS, a substantial proportion (51.4%) of patients needed to be referred for a LSM evaluation. eLIFT provided a satisfying trade-off between sensitivity and specificity, with acceptable performance, and only 33% of patients requiring LSM evaluation (S3 Table). Among the 34 patients addressed for LSM, five had suspected AdLF and three accepted liver biopsy; AdLF was histologically confirmed in these three patients. Results regarding NITs, LSM and TLB are summarized in S3 Fig.

### Liver biopsy

Transjugular liver biopsy (TLB) was proposed for the 10 patients with suspected AdLF on Fibroscan. Five patients consented to undergo TLB, and histological results were as follows: one patient had liver cirrhosis (F4 in METAVIR), two had severe fibrosis with septa (one patient had liver cirrhosis (F4 in METAVIR), two had severe fibrosis with septa (one patient had fibrosis staged F3 in METAVIR associated with histiocytic granuloma related to sarcoidosis and the other had fibrosis staged F3 according to the NASH CRN system), one patient had mild perisinusoidal/pericellular fibrosis (NASH CRN stage F1a), and the last patient had no fibrosis (F0 in METAVIR) but regenerative nodular hyperplasia. The number of liver fragments per biopsy varied from 1 to 4, the median length of fragments was 10 mm (range: 4 to 20) and the median number of portal tracts was 11 (range: 6–19). No complications occurred following TLB and all patients were discharged from hospital the day after TLB.

## Discussion

This prospective study was designed to detect AdLF in CAD patients using easily available NITs, since these patients usually share metabolic comorbidities with NASH. The main findings of the present study are as follows: 1) 5.3% of CAD patients had suspected AdLF on LSM evaluation. 2) When considering both the overall CAD population and the patients “at-risk” of NAFLD, eLIFT and NFS both provided a good trade-off between sensitivity and specificity, and could be used as the first-line screening test for AdLF. NPVs are high and not very different among NITs (between 94.3% and 97.4% in the whole population and between 93% and 96.5% in the “at-risk” group, excluding Forns, which is not appropriate in this study because this test leads to unnecessary investigations due to its too high positivity (84.6%)). However, high NPVs in unselected populations with low prevalence of AdLF are expected [7]. Indeed, even if all patients were classified as non-AdLF using NITs, the NPV would be 94.7% (179/189), which is not very different from the NPVs presented in our study. The proportion of patients referred for LSM (around 10–25%) seems to be adequate using APRI and FIB-4 but the sensitivity of these tests is very low (20–30%). NFS and eLIFT showed the most attractive performance with high NPV to confidently rule out AdLF, adequate proportions of referrals for LSM (38.5% for NFS and 31.2% for eLIFT) and acceptable sensitivity (70% for NFS and 60% for eLIFT) in the whole population. 3) the small subgroup of “high-risk” obese patients was too restrictive and failed to identify two out of 10 patients with LSM ≥ 8 kPa. Further studies with larger sample sizes are needed to draw more definite conclusions regarding performance of NITs on such subgroup of patients.

Liver fibrosis is the strongest predictor of cardiovascular events [10, 27], such as myocardial infarction and stroke, and of cardiovascular and all-cause mortality in patients with NAFLD [4, 11, 28, 29]. There is some data demonstrating that high liver fibrosis scores had a detrimental clinical impact on long-term outcomes in CAD patients, even when considering subgroup analysis regarding age, sex, arterial hypertension or diabetes [30]. Conversely, data regarding the prevalence of AdLF in the specific population of CAD patients are not available. There are many potential reasons for this lack of information, including the following possible hypotheses: 1) a lack of awareness of cardiologists regarding the strong negative relation between liver fibrosis and cardiovascular events, another heart-liver axis that less well recognized by cardiologists than portopulmonary hypertension, for example, which requires their intervention. 2) A lack of knowledge of non-patented easy-to-calculate NITs: in our university hospital, the main reason was a lack of time to screen for liver fibrosis, which was not recognized as a priority in the context of busy schedules with cardiovascular emergencies, even though many of these emergencies concern dysmetabolic patients at risk of developing AdLF, and consequently liver disease-related complications.

To the best of our knowledge, this is the first prospective study to evaluate the performance of NITs for systematic screening for AdLF in the specific population of CAD patients. FIB-4 appears to be ideally suited for use in the primary healthcare setting to identify NAFLD patients without AdLF, due to its excellent NPV [31, 32], its simplicity and its wide availability. Unfortunately, in our population of patients with CAD, FIB-4 had the lowest sensitivity (20%) of the five NITs. The reasons for the discordant results of FIB-4 between the general population and patients with CAD remain elusive. The variables associated with high/intermediate FIB-4 were different from those associated with LSM ≥ 8 kPa (S4A–S4C Table). In our study, two variables were found to be associated with LSM (namely HDL-cholesterol and GGT), but neither of them is included in the FIB-4 formula (similarly, those variables are not incorporated in the NFS formula). The fact that GGT is included in the eLIFT score may explain its superior performance compared to FIB-4. In addition, nearly 25% of patients were in the intermediate zone of FIB-4, consequently increasing the false positive results and decreasing the PPV (only 4.2%). We also need to bear in mind that FIB-4 and other NITs were mainly developed in chronic viral hepatitis to diagnose significant liver fibrosis [14]. Consequently, different performances with these NITs can be expected when used in other specific populations with low prevalence of AdLF, like our CAD patients.

We also observed that the percentage of patients with results classing them in the high-risk zone of FIB-4, Forns, APRI and NFS was low (between 0.5% and 13.6%), requiring the aggregation of the intermediate- and high-risk zones to avoid failing to identify patients who had suspected AdLF on Fibroscan. The main drawback of such aggregation is an unavoidable increase in the number of false positive results of NITs, but with the advantage of simplifying decision-making by proposing a single cut-off separating “high” and “low” zones (like eLIFT).

We found a prevalence of elevated LSM ≥8 kPa of 5% in our cohort of patients with CAD, which is very close to rates previously observed in the general population [33–35]. A recent study reported that the sensitivity of FIB-4 and NFS for the case-finding of LSM ≥8 kPa in general population cohorts was respectively 37% and 52% [36], which is not very different from our results. In fact, patients with LSM ≥8 kPa in our study were all included in the subgroup of “at-risk NAFLD” (i.e., patients with elevated ALT, diabetes, dyslipidemia or BMI ≥25 kg/m^2^), reinforcing the concept of targeted AdLF case-finding in patients with liver-risk factors, rather than non-targeted screening procedures in supposed at-risk populations such as CAD patients [36]. Future studies in larger samples of CAD patients are required to confirm this finding.

To improve the performance of NITs, we stratified our population into subgroups with a higher risk of NAFLD (namely “at risk” and “at high-risk” of NAFLD). In patients “at risk” of NAFLD (i.e., overweight or obese patients), no cases of suspected AdLF on Fibroscan were missed, the performance of NITs improved slightly, and the number of patients referred for LSM was smaller. Unfortunately, by narrowing the initial population further to a group considered at "high-risk” of NAFLD (i.e., obese patients), we missed two patients with AdLF on Fibroscan. Limiting screening for liver fibrosis in CAD patients to those with some components of the metabolic syndrome is supported by the physiopathology, implying lipotoxic factors that are pathogenically involved in atherogenesis and consequently in CAD. However, the underlying mechanisms by which NAFLD increases the risk of CVD have not been well established; they are complex and may involve insulin resistance (associated with high serum levels of oxidized-LDL and glycated-LDL), systemic inflammation, oxidative stress, neuroendocrine activation (renin-angiotensin system and sympathetic nervous system may exacerbate cardiac remodeling), imbalance of procoagulant and anticoagulant factors, hepatokine imbalance, which can induce metabolic abnormalities, dysbiosis of the gut microbiota and genetics [2, 37, 38]. A better understanding of the “crosstalk” between NAFLD and CVD will help to develop innovative therapeutic strategies, such as targeting oxidized phospholipids to improve both NASH and atherosclerosis [39, 40].

Our study suffers from several limitations, notably the small sample size (n = 199), the single-centre design and the small number of patients (n = 10) with suspected AdLF on Fibroscan. We acknowledge that our study was not sufficiently powered to draw definite conclusions regarding the most appropriate NITs to apply in CAD patients. Nevertheless, the major strengths of our proof-of-concept study include the prospective recruitment of “real-world” CAD patients and the innovative purpose. We are confident that our study will be replicated by larger, multicenter, prospective studies to validate our findings. Another limitation could be the lack of use of patented NITs (such as the Fibrotest^®^, Fibrometer^®^, ELF^™^ score and Hepascore), which have slightly improved diagnostic accuracy compared to non-patented NITs [41, 42]. Additional use of patented NITs in a two-step strategy could be useful to improve the selection of patients with suspected AdLF for referral to hepatological evaluation [43]. In the present study, we used only non-patented NITs for large-scale, routine clinical screening considering the availability of free online calculators and the easy of calculation for the cardiologists. Furthermore, there is room for debate in the interpretation of the results, which depend on the capacity of LSM evaluation to detect the presence of AdLF. Nevertheless, LSM evaluations in our study were performed by two experienced operators who applied existing validity criteria [14], and statistical analyses were performed only in patients with a valid LSM. Another limitation is the detection of hepatic steatosis using the cut-off value of CAP (275 dB/m) reported by the recent EASL guidelines [22]. Indeed, this cut-off is not consensual since it may vary greatly according to the population involved (pediatric [44, 45] or adult [46–48]) and depending on the desired objective (to maximize the sum of sensitivity and specificity, achieve greater accuracy, etc.). Recent data demonstrated that simple steatosis is not prognostically as benign as previously thought [49, 50] and, consequently, early detection of NAFLD using reliable non-invasive and widely available techniques will be a relevant task in the future. Finally, only half of the patients with suspected AdLF underwent liver biopsy; however, the proportion of patients who consented to liver biopsy in other similar studies is also low, at 31% and 4% in the studies by Caballeria et al. [35] and Poynard et al. [51], respectively. Although liver biopsy is still considered as the reference for the diagnosis of liver fibrosis, this procedure remains costly and invasive (especially in CAD patients taking anticoagulants and/or antiplatelet drugs) and cannot be used to evaluate liver fibrosis in the framework of large-scale screening. Liver biopsy is sometimes associated with rare but potentially life-threatening complications, even when using the transjugular route. In our study, we used TLB, since we considered our patients to be at high risk for bleeding [52]. The reported complication rate with TLB is 7.1%, major complications (like intraperitoneal hemorrhage or pneumothorax) are extremely rare (0.5%) and deaths are exceptional (0.09%) [52]. We did not observe any complications in the five patients who had TLB in our study.

## Conclusion

Our findings indicate that nearly 5% of patients aged 18–70 years with proven CAD had advanced liver fibrosis, as assessed by LSM ≥ 8 kPa. We propose the pragmatic use of first-line non-invasive biochemical liver fibrosis tests to adequately rule out CAD patients who require no further assessment (e.g. by Fibroscan, then liver biopsy). The most accurate and convenient way to refer CAD patients to hepatologists for secondary evaluation combines the identification of overweight/obese patients (with or without hypertriglyceridemia, diabetes, elevated ALT) and next, the calculation of NITs. NFS and eLIFT had similar and good diagnostic performances, but only eLIFT can be calculated at the bedside in a straightforward way without a computer. However, the low number of CAD patients with AdLF included in our study precludes drawing any definite conclusion about which are the most appropriate NITs for cardiologists. We are confident that further, larger and well-powered studies will soon contribute to this debate.

## Supporting information

S1 Fig Screening for advanced liver fibrosis using non-invasive fibrosis tests in the whole population. In the initial population (n = 199), 10 patients had LSM ≥ 8 kPa and 5 of them consented to undergo a transjugular liver biopsy (TLB); 3 patients were F3/F4 and 2 had no liver fibrosis (F0). Missing data (MD) are reported for each test. H-I, High and Intermediate zones; LSM, Liver stiffness measurement in Kpa.
(DOCX)

S2 Fig Screening for advanced liver fibrosis using non-invasive fibrosis tests in the group “at risk” of NAFLD. As in the initial population (n = 199), 10 patients in the group “at risk” for NAFLD (n = 158) had LSM ≥ 8 kPa and 5 of them consented to undergo transjugular liver biopsy (TLB); 3 patients were F3/F4 and 2 had no liver fibrosis (F0). Missing data (MD) are reported for each test. H-I, High and Intermediate zones; LS, Liver stiffness measurement in Kpa.
(DOCX)

S3 Fig Screening for advanced liver fibrosis using non-invasive fibrosis tests in the group “at high-risk” of NAFLD. In the group “at high-risk” of NAFLD (n = 111), 8 patients had LSM ≥ 8 kPa and 3 of them consented to undergo transjugular liver biopsy (TLB); all 3 were F3/F4. Missing data (MD) are reported for each test. H-I, High and Intermediate zones; LSM, Liver stiffness measurement in Kpa.
(DOCX)

S1 Table Variables associated with liver stiffness measurement (LSM) by multivariate analysis. Candidate variables for inclusion in the multivariate model (ALT or AST, GGT and HDL cholesterol) were selected based on the results of univariate analysis (Table 2). Continuous variables were dichotomized according to their best thresholds, as determined by Youden’s index, to discriminate low (< 8 kPa) and high (≥ 8 kPa) LSM in the whole population of patients with valid LSM (n = 189). The cutoffs used for GGT and HDL were as follows: GGT < or ≥ 68 IU/L, HDL < or ≥ 0.91 mmol/L. AST or ALT (used separately in different models) were not significant when dichotomized according to their best thresholds.
(DOCX)

S2 Table Proportion of patients in the different zones of non-invasive fibrosis tests. Proportions of patients in the different zones of NITs were calculated in the whole population (n = 199). For all analyses, we combined the intermediate- and high-risk zones of NITs because the proportion of patients in the high-risk zones was small, ranging from 0.5% (APRI) to 13.6% (Forns). AdLF, advanced liver fibrosis; H-I zone, high and intermediate zones; LSM, liver stiffness measurement; NITs, non-invasive fibrosis tests.
(DOCX)

S3 Table Screening for advanced liver fibrosis using non-invasive fibrosis tests in the “high-risk” group of NAFLD^#^ with a valid fibroscan. &: High and intermediate zones (H-I zones) of NFS, APRI, Forns and Fib-4 have been combined; patients in the H-I zones and those with eLIFT ≥ 8 had suspected AdLF and were referred for a liver stiffness measurement. #: Patients in the “high-risk” group of NAFLD had at least one of the following conditions: elevated ALAT (n = 16, 14.4%), diabetes (n = 59, 53.1%), triglycerides ≥ 1.7 mmol/L (n = 57, 51.3%), BMI ≥ 30 kg/m^2^ (n = 53, 47.7%). AdLF, advanced liver fibrosis; LSM, liver stiffness measurement; NITs, non-invasive fibrosis tests; Se, sensitivity; Spe, specificity; NPV, negative predictive value; PPV, positive predictive value.
(DOCX)

S4 Table **(A-C)** Variables associated with high/intermediate FIB-4 by univariate analysis **(A)**, by multivariate analysis using median values of variables **(B)** and Youden’s index **(C)**. **S4A**: Data were analyzed among the 189 patients with a valid Fibroscan and are expressed in median with IQR or mean (± standard deviation). By univariate analysis, there was no difference between groups regarding C-reactive protein, creatinine, total cholesterol, HDL and LDL-cholesterol, glycaemia, obesity, arterial hypertension or metabolic syndrome. H-I, High and Intermediate zones. **S4B**: Quantitative variables dichotomized according to their median values: platelets < or ≥ 246 G/L, AST < or ≥ 26 IU/L, ALT < or ≥ 28 IU/L and leukocytes < or ≥ 7.86 G/L. **S4C**: Quantitative variables dichotomized according to the best threshold (Youden’s index): platelets < or ≥ 230 G/L, AST < or ≥ 33 IU/L, ALT < or ≥ 15 /L and leukocytes < or ≥ 7.07 G/L.
(DOCX)

S1 Data (XLSX)

S1 File Non-invasive fibrosis tests used in CoroNASH study. (DOCX)

S2 File Assessment of chronic liver disease during Hepatology consultation. (DOCX)

## Abbreviations

AdLF: advanced liver fibrosis
APRI: AST-to platelet ratio index
BMI: body mass index
CAD: coronary artery disease
CAP: controlled attenuation parameter
CVD: cardiovascular disease
eLIFT: easy liver fibrosis test
FIB-4: Fibrosis-4
LSM: liver stiffness measurement
NAFLD: nonalcoholic fatty liver disease
NASH: Nonalcoholic steatohepatitis
NFS: NAFLD fibrosis score
NITs: non-invasive fibrosis tests
TLB: transjugular liver biopsy
