# Supplementary material for: Systematic screening for advanced liver fibrosis in patients with coronary artery disease: The CORONASH study
Source: PLoS One. 2022 May 26;17(5):e0266965. doi: 10.1371/journal.pone.0266965 (PMC9135299; doi:10.1371/journal.pone.0266965)
Supplement: S1 Fig — In the initial population (n = 199), 10 patients had LSM ≥ 8 kPa and 5 of them consented to undergo a transjugular liver biopsy (TLB); 3 patients were F3/F4 and 2 had no liver fibrosis (F0). Missing data (MD) are reported for each test. H-I, High and Intermediate zones; LSM, Liver stiffness measurement in Kpa. (DOCX) [file pone.0266965.s001.docx]

**Figure S1: Screening for advanced liver fibrosis using non-invasive fibrosis tests in the whole population**

**
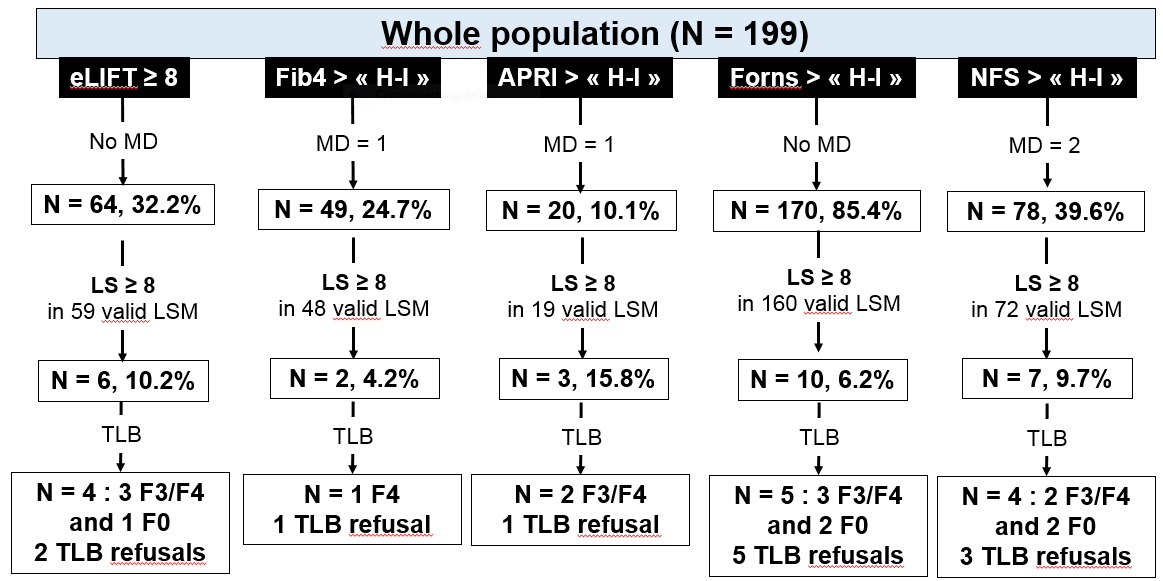
**
