# Supplementary material for: Systematic screening for advanced liver fibrosis in patients with coronary artery disease: The CORONASH study
Source: PLoS One. 2022 May 26;17(5):e0266965. doi: 10.1371/journal.pone.0266965 (PMC9135299; doi:10.1371/journal.pone.0266965)
Supplement: S2 Fig — As in the initial population (n = 199), 10 patients in the group “at risk” for NAFLD (n = 158) had LSM ≥ 8 kPa and 5 of them consented to undergo transjugular liver biopsy (TLB); 3 patients were F3/F4 and 2 had no liver fibrosis (F0). Missing data (MD) are reported for each test. H-I, High and Intermediate zones; LS, Liver stiffness measurement in Kpa. (DOCX) [file pone.0266965.s002.docx]

**Figure S2: Screening for advanced liver fibrosis using non-invasive fibrosis tests in the group “at risk” of NAFLD**

**
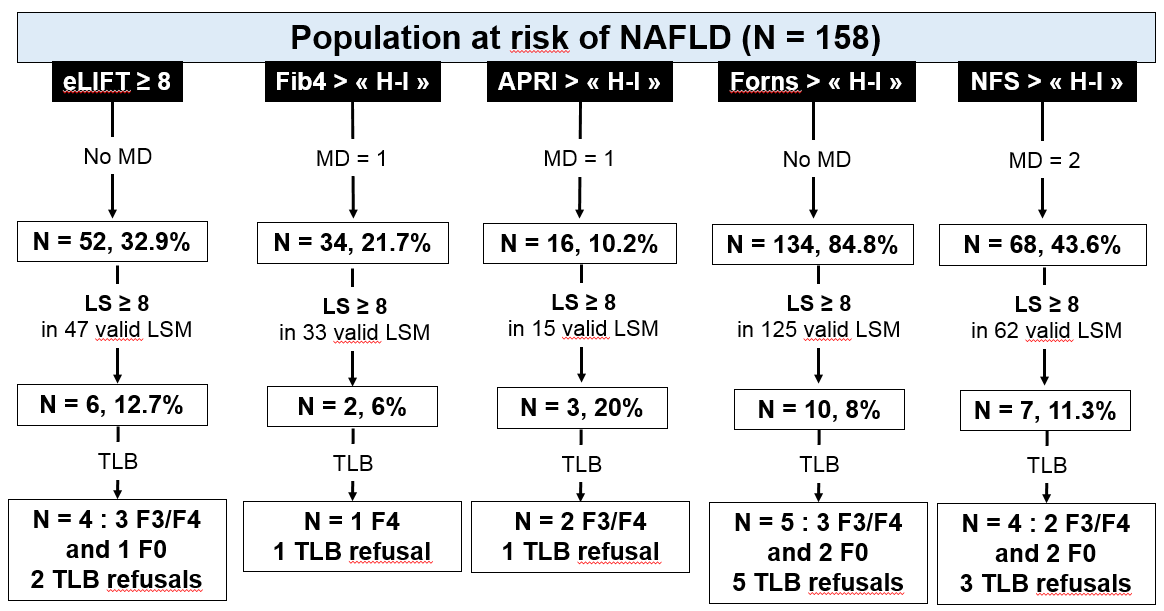
**
