# Supplementary material for: Systematic screening for advanced liver fibrosis in patients with coronary artery disease: The CORONASH study
Source: PLoS One. 2022 May 26;17(5):e0266965. doi: 10.1371/journal.pone.0266965 (PMC9135299; doi:10.1371/journal.pone.0266965)

**Figure S3: Screening for advanced liver fibrosis using non-invasive fibrosis tests in the group “at high-risk” of NAFLD**


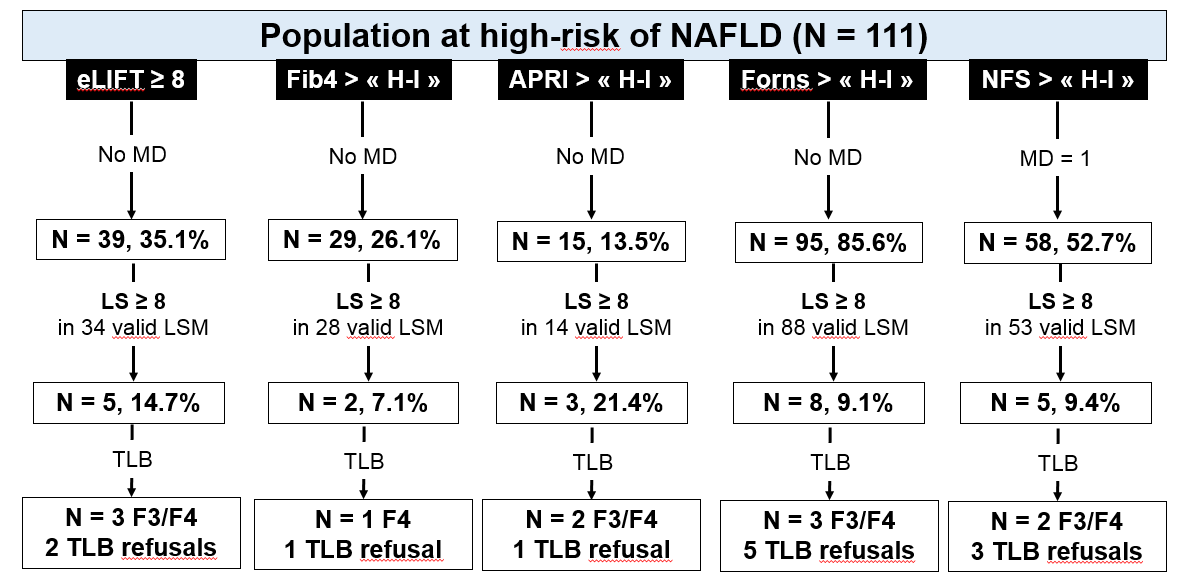

Supplement: S3 Fig — In the group “at high-risk” of NAFLD (n = 111), 8 patients had LSM ≥ 8 kPa and 3 of them consented to undergo transjugular liver biopsy (TLB); all 3 were F3/F4. Missing data (MD) are reported for each test. H-I, High and Intermediate zones; LSM, Liver stiffness measurement in Kpa. (DOCX) [file pone.0266965.s003.docx]
