# Supplementary material for: Systematic screening for advanced liver fibrosis in patients with coronary artery disease: The CORONASH study
Source: PLoS One. 2022 May 26;17(5):e0266965. doi: 10.1371/journal.pone.0266965 (PMC9135299; doi:10.1371/journal.pone.0266965)
Supplement: S1 Table — Candidate variables for inclusion in the multivariate model (ALT or AST, GGT and HDL cholesterol) were selected based on the results of univariate analysis (Table 2). Continuous variables were dichotomized according to their best thresholds, as determined by Youden’s index, to discriminate low (< 8 kPa) and high (≥ 8 kPa) LSM in the whole population of patients with valid LSM (n = 189). The cutoffs used for GGT and HDL were as follows: GGT < or ≥ 68 IU/L, HDL < or ≥ 0.91 mmol/L. AST or ALT (used separately in different models) were not significant when dichotomized according to their best thresholds. (DOCX) [file pone.0266965.s004.docx]

**Table S1: Variables associated with liver stiffness measurement (LSM) by multivariate analysis**

| **Multivariate analysis** | **OR** | **95% CI** | **P** |
| --- | --- | --- | --- |
| **GGT** | 9.03 | 2.21-36.94 | 0.002 |
| **HDL cholesterol** | 0.14 | 0.03-0.63 | 0.01 |
