# Supplementary material for: Systematic screening for advanced liver fibrosis in patients with coronary artery disease: The CORONASH study
Source: PLoS One. 2022 May 26;17(5):e0266965. doi: 10.1371/journal.pone.0266965 (PMC9135299; doi:10.1371/journal.pone.0266965)
Supplement: S2 Table — Proportions of patients in the different zones of NITs were calculated in the whole population (n = 199). For all analyses, we combined the intermediate- and high-risk zones of NITs because the proportion of patients in the high-risk zones was small, ranging from 0.5% (APRI) to 13.6% (Forns). AdLF, advanced liver fibrosis; H-I zone, high and intermediate zones; LSM, liver stiffness measurement; NITs, non-invasive fibrosis tests. (DOCX) [file pone.0266965.s005.docx]

**Table S2: Proportion of patients in the different zones of non-invasive fibrosis tests**

| **Variables** | **Suspected AdLF on NITs, n (%)** | **LSM ≥ 8 kPa in patients with a suspected AdLF on NITs, n (%)** |
| --- | --- | --- |
| **NFS > H-I zone**  **NFS > high zone** | 78 (39.6)  11 (5.6) | 7 (9.0)  1 (9.1) |
| **Forns > H-I zone**  **Forns > high zone** | 170 (85.4)  27 (13.6) | 10 (5.9)  4 (14.0) |
| **APRI > H-I zone**  **APRI > high zone** | 20 (10.1)  1 (0.5) | 3 (15)  1 (100) |
| **FIB-4 > H-I zone**  **FIB-4 > high zone** | 49 (24.8)  5 (2.5) | 2 (4.1)  1 (20) |
| **eLIFT ≥ 8** | 64 (32.2) | 6 (9.4) |
