# Supplementary material for: Systematic screening for advanced liver fibrosis in patients with coronary artery disease: The CORONASH study
Source: PLoS One. 2022 May 26;17(5):e0266965. doi: 10.1371/journal.pone.0266965 (PMC9135299; doi:10.1371/journal.pone.0266965)
Supplement: S3 Table — &: High and intermediate zones (H-I zones) of NFS, APRI, Forns and Fib-4 have been combined; patients in the H-I zones and those with eLIFT ≥ 8 had suspected AdLF and were referred for a liver stiffness measurement. #: Patients in the “high-risk” group of NAFLD had at least one of the following conditions: elevated ALAT (n = 16, 14.4%), diabetes (n = 59, 53.1%), triglycerides ≥ 1.7 mmol/L (n = 57, 51.3%), BMI ≥ 30 kg/m2 (n = 53, 47.7%). AdLF, advanced liver fibrosis; LSM, liver stiffness measurement; NITs, non-invasive fibrosis tests; Se, sensitivity; Spe, specificity; NPV, negative predictive value; PPV, positive predictive value. (DOCX) [file pone.0266965.s006.docx]

**Table S3: Screening for advanced liver fibrosis using non-invasive fibrosis tests in the “high-risk” group of NAFLD^#^ with a valid fibroscan**

| **Tests** | **N^#^** | **Se**  **% (IC 95%)** | **Spe**  **% (IC 95%)** | **PPV %**  **(IC 95%)** | **NPV %**  **(IC 95%)** | **Accuracy**  **%** | **AUROC**  **(IC 95%)** | **Patients with suspected AdLF on NITs^&^, n (%)** | **LSM ≥ 8 kPa among H-I NITs^&^, n (%)** |
| --- | --- | --- | --- | --- | --- | --- | --- | --- | --- |
| **NFS** | 103 | 62.5  (30.6-86.3) | 49.5  (39.6-59.3) | 9.4  (3.8-15.0) | 94.0  (89.4-98.6) | 50.5  (45.6-55.4) | 0.650  (0.387-0.814) | 53 (51.4) | 5 (9.4) |
| **Forns** | 104 | 100  (67.5-100) | 16.7  (10.3-25.3) | 9.1  (3.6-14.6) | 100  (1-1) | 23.1  (19.0-27.2) | 0.819  (0.682-0.900) | 88 (84.6) | 8 (9.1) |
| **APRI** | 104 | 37.5  (13.7-69.4) | 88.5  (80.6-93.5) | 21.4  (13.5-29.2) | 94.4  (90.0-98.8) | 84.6  (81.1-88.1) | 0.791  (0.565-0.907) | 14 (13.5) | 3 (21.4) |
| **Fib4** | 104 | 25.0  (7.2-59.0) | 72.9  (63.3-80.8) | 7.1  (2.2-12.0) | 92.1  (86.9-97.3) | 69.2  (64.7-73.7) | 0.718  (0.532-0.838) | 28 (26.9) | 2 (7.1) |
| **eLIFT** | 104 | 62.5  (30.6-86.3) | 69.8  (60.0-78.0) | 14.7  (7.9-21.5) | 95.7  (91.8-99.6) | 69.2  (64.7-73.7) | 0.729  (0.477-0.869) | 34 (32.7) | 5 (14.7) |
