# Supplementary material for: Systematic screening for advanced liver fibrosis in patients with coronary artery disease: The CORONASH study
Source: PLoS One. 2022 May 26;17(5):e0266965. doi: 10.1371/journal.pone.0266965 (PMC9135299; doi:10.1371/journal.pone.0266965)
Supplement: S4 Table — (A-C) Variables associated with high/intermediate FIB-4 by univariate analysis (A), by multivariate analysis using median values of variables (B) and Youden’s index (C). S4A: Data were analyzed among the 189 patients with a valid Fibroscan and are expressed in median with IQR or mean (± standard deviation). By univariate analysis, there was no difference between groups regarding C-reactive protein, creatinine, total cholesterol, HDL and LDL-cholesterol, glycaemia, obesity, arterial hypertension or metabolic syndrome. H-I, High and Intermediate zones. S4B: Quantitative variables dichotomized according to their median values: platelets < or ≥ 246 G/L, AST < or ≥ 26 IU/L, ALT < or ≥ 28 IU/L and leukocytes < or ≥ 7.86 G/L. S4C: Quantitative variables dichotomized according to the best threshold (Youden’s index): platelets < or ≥ 230 G/L, AST < or ≥ 33 IU/L, ALT < or ≥ 15 /L and leukocytes < or ≥ 7.07 G/L. (DOCX) [file pone.0266965.s007.docx]

**Tables S4A-S4C: Variables associated with high/intermediate FIB-4 by univariate analysis (A), by multivariate analysis using median values of variables (B) and Youden’s index (C)**

**S4A.**

| **Univariate analysis** | **Patients with H-I FIB-4, N = 48** | **Patients with low FIB-4, N = 140** | **P** |
| --- | --- | --- | --- |
| **Age, yrs** | 60.5 ± 5.0 | 60.6 ± 7.9 | 0.29 |
| **BMI, kg/m²** | 26.7 ± 3.9 | 27.9 ± 4.4 | 0.10 |
| **BMI ≥ 25 kg/m², %** | 60.4 | 75.0 | 0.054 |
| **Alcohol consumption, g/d** | 10 [0-20] | 0 [0-10] | 0.026 |
| **Any alcohol consumption, n%** | 35.5 | 64.5 | 0.009 |
| **AST, IU/L** | 33.3 ± 19.5 | 26.5 ± 8.0 | 0.051 |
| **ALT, IU/L** | 25 [13-34] | 28 [21-37] | 0.055 |
| **GGT, IU/L** | 26 [19-55] | 31 [22-50] | 0.23 |
| **Platelets, G/L** | 205 ± 53 | 260 ± 69 | <0.0001 |
| **Prothrombin index or Factor V, %** | 94.3 ± 10.1 | 96.4 ± 12.1 | 0.23 |
| **Leukocytes, G/L** | 7.1 ± 1.9 | 8.4 ± 2.2 | 0.0001 |

| **Variables** | **OR** | **95% CI** | **P** |
| --- | --- | --- | --- |
| **Platelets** | 0.13 | 0.05-0.32 | < 0.0001 |
| **ALT** | 0.12 | 0.04-0.38 | 0.0002 |
| **AST** | 4.19 | 1.48-12.13 | 0.008 |
| **Leukocytes** | 0.48 | 0.21-1.08 | 0.07 |
| **Any alcohol consumption** | 2.25 | 1.05-4.84 | 0.04 |

**S4B.**

**S4C.**

| **Variables** | **OR** | **95%CI** | **P** |
| --- | --- | --- | --- |
| **Platelets** | 0.13 | 0.05-0.33 | < 0.0001 |
| **ALT** | 0.03 | 0.01-0.14 | < 0.0001 |
| **AST** | 5.96 | 2.08-17.08 | 0.0008 |
| **Leukocytes** | 0.19 | 0.07-0.53 | 0.0009 |
| **Any alcohol consumption** | 4.09 | 1.60-10.46 | 0.003 |
