# Supplementary material for: Systematic screening for advanced liver fibrosis in patients with coronary artery disease: The CORONASH study
Source: PLoS One. 2022 May 26;17(5):e0266965. doi: 10.1371/journal.pone.0266965 (PMC9135299; doi:10.1371/journal.pone.0266965)
Supplement: S1 File — (DOCX) [file pone.0266965.s009.docx]

**File S1: Non-invasive fibrosis tests used in CoroNASH study**

Five non-invasive fibrosis tests (NITs: APRI, FIB-4, Forns, NFS, and eLIFT) were used to evaluate liver fibrosis and are summarized below:

- AST to Platelet Ratio (APRI) = AST (/ULN)/platelet (10^9^/L) x 100
- FIB-4 = age (years) x AST [U/L]/(platelets [10^9^/L] x (ALT [U/L])^1/2^
- Forns index = 7.811 – 3.131 x ln (platelets (10^9^/L)) + 0.781 x ln (GGT (UI/L)) + 3.467 x ln (age (years)) – 0.014 x (total cholesterol (mg/dL))
- NAFLD Fibrosis Score (NFS) = (-1.675 + 0.037 x age (years) + 0.094 x BMI (kg/m^2^) + 1.13 x IFG/diabetes (yes = 1, no = 0) + 0.99 x AST/ALT ratio - 0.013 x platelet count (x10^9^/L) - 0.66 x albumin [g/dl])
- eLIFT score

| **ITEM** | **POINTS** |
| --- | --- |
| **Age** (year)  ≥ 40 | 3 |
| **AST** (IU/L)  35-69  ≥ 70 | 2  4 |
| **Male sex** | 1 |
| **GGT** (IU/L)  35-89  ≥ 90 | 1  2 |
| **Platelets** (G/L)  170-249  < 170 | 1  4 |
| **Prothrombin time** (%)  84-96  < 84 | 2  4 |

We categorized patients into low-, intermediate- and high-risk groups for AdLF based on the following suggested (lower and upper) cutoffs: APRI (0.5 and 1.5), FIB-4 (lower cutoff 1.30 if age <65, and 2.0 if age ≥65 years; upper cutoff 2.67), Forns (4.2 and 6.9), NFS (lower cutoff -1.455 if age <65 years and 0.12 if age ≥65; upper cutoff 0.676). The eLIFT was considered negative when the score was < 8 and positive when ≥ 8.
