# Supplementary material for: Systematic screening for advanced liver fibrosis in patients with coronary artery disease: The CORONASH study
Source: PLoS One. 2022 May 26;17(5):e0266965. doi: 10.1371/journal.pone.0266965 (PMC9135299; doi:10.1371/journal.pone.0266965)
Supplement: S2 File — (DOCX) [file pone.0266965.s010.docx]

**File S2: Assessment of chronic liver disease during Hepatology consultation**

Patients with suspected AdLF on Fibroscan (liver stiffness ≥ 8 kPa) were referred for ambulatory hepatology consultation, with the results of blood samples performed in the workup of these patients to rule out specific chronic liver diseases*.* During this consultation, the hepatologist (TT, DW, CR, JPC and CV) looked mainly for excessive alcohol consumption, components of the metabolic syndrome (BMI, arterial hypertension, diabetes, dyslipidemia) and common risk factors for hepatitis. Blood samples included hepatitis B and C markers (hepatitis B surface antigen, anti-HBc and anti-HBs antibodies, hepatitis C antibody), glucose and glycosylated hemoglobin, lipids (total cholesterol, triglycerides, HDL- and LDL-cholesterol), ferritin, transferrin saturation coefficient, alpha-1 antitrypsin, ceruloplasmin, liver-related autoantibodies (antinuclear antibodies [ANA], smooth muscle antibodies [SMA] and antimitochondrial antibodies [AMA]).

Among the 10 patients with LSM ≥ 8 kPa, only one had excessive alcohol consumption (alcohol consumption 60 g/day) together with metabolic syndrome. All patients were seronegative for hepatitis B surface antigen and hepatitis C antibodies, six patients had both anti-HBc and anti-HBs antibodies and only one carried isolated anti-HBc antibodies. Four patients had a serum ferritin > 204 ng/mL (i.e., the upper limit of normal laboratory range) but all of them had a transferrin saturation coefficient lower than 45%. None of these patients had alpha-1 antitrypsine < 0.9 g/L (i.e., the lower limit of normal laboratory range) and all of these patients had normal serum ceruloplasmin level. Four patients had non-specific ANA (titers at 1:80 and 1:160 for 3 and 1 patients, respectively) and another patient had isolated SMA (titer at 1:40) with normal serum transaminase levels. Autoantibodies (ANA, SMA and AMA) were detected on indirect immunofluorescence for routine testing.
